# Surgical treatment for secondary aortoesophageal fistula after prosthetic aortic replacement: A report of four cases

**DOI:** 10.1016/j.ijscr.2020.08.021

**Published:** 2020-08-29

**Authors:** Masahide Enomoto, Takeshi Kinoshita, Noriyuki Takashima, Fumihiro Miyashita, Tomoaki Suzuki

**Affiliations:** Department of Cardiovascular Surgery, Shiga University of Medical Science, Shiga, Japan

**Keywords:** Aortoesophageal fistula, Total arch replacement, Descending aortic replacement, Thoracic endovascular aortic repair, Aortic graft infection, Esophagectomy

## Abstract

**Introduction:**

With the increase of thoracic aortic aneurysm surgery and thoracic endovascular aortic repair, secondary aortoesophageal fistula (AEF) has been reported. However, the treatment strategy for AEF remains controversial.

**Presentation of cases:**

Four patients who had undergone prosthetic aortic replacement for thoracic aortic aneurysm 4–36 months previously, were hospitalized with sepsis-like symptoms. They were diagnosed with aortic prosthetic graft infection after computed tomography revealed ectopic gas around the prosthesis. After that, esophagogastroduodenoscopy revealed an esophageal perforation, so we diagnosed AEF. They received medication and stepwise surgery; 1 patient was discharged, 2 remain hospitalized, and 1 died.

**Discussion:**

Some reports have suggested that combined surgery provides better outcomes for AEF. Infection may be controlled by esophagectomy and antibiotic treatment, so prosthesis replacement is not always necessary. However, we should note that infection between a prosthetic graft and the native aorta brings a danger of pseudoaneurysm of the anastomosis.

**Conclusion:**

Based on our experience we conclude that surgery performed stepwise along with infection control and general health improvement is a valid treatment strategy for secondary AEF after prosthetic aortic replacement.

## Highlights

• Secondary aortoesophageal fistula (AEF) after aortic prosthetic replacement is rare, so there have been few reports assembling multiple cases.
• AEF is difficult to diagnose early if there is little experience, and hence is associated with high mortality and morbidity rates.
• The strategy involving stepwise surgery in coordination with infection control and general health improvement was attempted in our facility.
• From our observations, this approach appears to be a valid strategy for secondary AEF after aortic prosthetic replacement.

## 1 Introduction

Aortoesophageal fistula (AEF) may be primary or secondary. With the increase of thoracic aortic aneurysm surgery and thoracic endovascular aortic repair (TEVAR), secondary AEF has been reported [[1], [2], [3]]. Some treatment strategies for AEF have been presented [[4], [5], [6], [7], [8], [9]]. However, there have been few reports with multiple cases of secondary AEF after prosthetic aortic replacement, so the treatment strategies remain controversial. In our facility, we have attempted a strategy including performing surgery stepwise for 4 cases of AEF after prosthetic aortic replacement, and we herein report them. This work has been reported in line with the PROCESS criteria [10].

## 2 Case presentation

### 2.1 Brief case histories before staged surgeries

Case 1: an 83-year-old man. Around 28 months after total arch replacement (TAR), he suffered fever and shivering. Prosthetic graft infection was diagnosed when computed tomography (CT) showed ectopic gas around the prosthesis. After 17 days in hospital, an infected pseudoaneurysm at the distal anastomosis of TAR and a resulting aortobronchial fistula were found. Emergency descending aorta replacement (DAR) was performed, and he was saved. Nine days after DAR, an air leak from the chest drain was found, and AEF was confirmed 2 days later by esophagogastroduodenoscopy (EGD).

Case 2: a 72-year-old man. Four months after TAR, he suffered fever and shivering. Prosthetic graft infection was diagnosed by CT. After 10 days in hospital a diagnosis of AEF was confirmed by EGD.

**Fig. 1:**
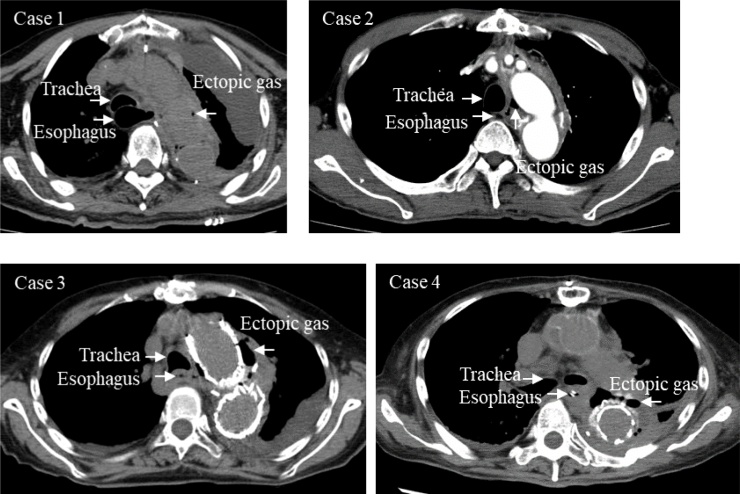
Computed tomography of the chest revealed ectopic gas around the prosthesis, which appears continuous from the esophagus.

**Fig. 2:**
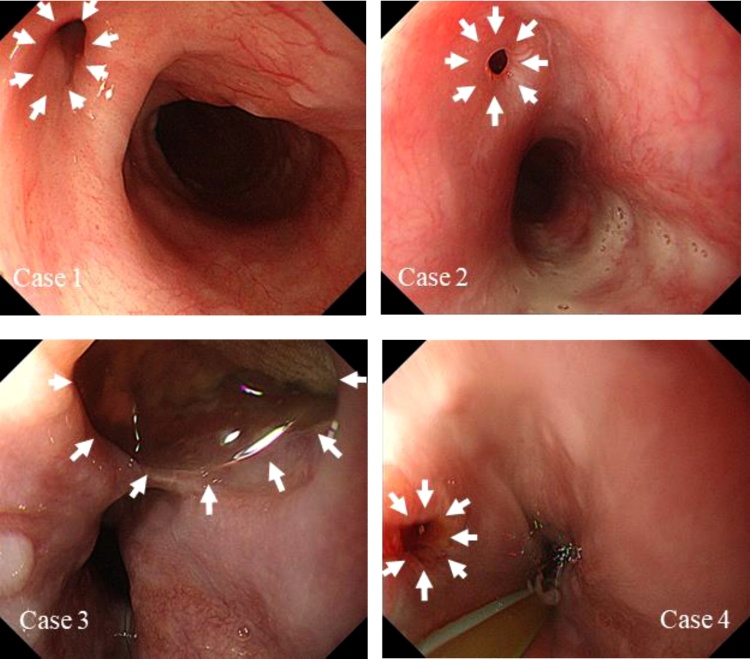
Esophagogastroduodenoscopy showed an aortoesophageal fistula (white arrows) in the esophagus.

Case 3: an 88-year-old woman. Around 36 months after TAR, a pseudoaneurysm at the distal anastomosis of TAR and a resulting left main bronchial obstruction occurred. Emergency TEVAR was performed, and she was saved. She developed a fever 18 days after TEVAR, and prosthetic graft infection was diagnosed by CT (Fig. 1). A diagnosis of AEF was confirmed by EGD (Fig. 2), 31 days after TEVAR.

Case 4: a 79-year-old woman. Around 17 months after DAR, she had fever, and prosthetic graft infection was diagnosed by CT. On her 2nd day in hospital, an infected pseudoaneurysm at the proximal site of DAR and a resulting aortobronchial fistula were found. Emergency TAR was performed, and she was saved, but 48 days later she suffered fever and shivering. Reoccurrence of prosthetic graft infection was diagnosed by CT, and AEF was confirmed by EGD 50 days after TAR.

Detailed description of our cases with AEF are shown in Table 1.

**Table 1:** Detailed description of our cases with aortoesophageal fistula.

Case	1	2	3	4
Age	83	72	88	79
Sex	male	male	female	female
Fistula mechanism	secondary	secondary	secondary	secondary
Site of fistula	middle thoracic esophagus (28 cm from incisors)	middle thoracic esophagus (30 cm from incisors)	middle thoracic esophagus (28 cm from incisors)	middle thoracic esophagus (28 cm from incisors)
Previous procedure	TAR (previous operation) DAR (this hospitalization)	TAR (previous operation)	TAR (previous operation) TEVAR (this hospitalization)	DAR (previous operation) TAR (this hospitalization)
Initial symptoms	air leak from drain	fever, shivering	fever	fever, shivering
Times till symptoms	28 months after TAR 9 days after DAR	4 months after TAR	36 months after TAR 18 days after TEVAR	17 months after DAR 50 days after TAR
Interval from initial symptoms to diagnosis	2 days	10 days	13 days	2 days
Interval from diagnosis to first operation	0 day	2 days	1 day	1 day
First-stage	esophagectomy	esophagectomy	esophagectomy	esophagectomy
Result	survival	survival	survival	survival
Interval between stages	–	37 days	–	28 days
Second-stage	–	TEVAR	–	Redo DAR
Result	survival	survival	survival	survival
Interval between stages	86 days	61 days	103 days	79 days
Third-stage	esophageal reconstruction	esophageal reconstruction	esophageal reconstruction	treatment of infection and DIC
result	survival	survival	survival	
Interval between stages	109 days	117 days	81 days	
Fourth-stage	death	discharge	waiting for closure of cutaneous fistula	

TAR: total arch replacement; DAR: descending aorta replacement; TEVAR: thoracic endovascular aortic repair; DIC: disseminated intravascular coagulation.

### 2.2 Our staged treatment strategy is as follows

First-stage surgery is esophagectomy, esophagostomy, and enterostomy. The first operation involves resection of the esophagus, formation of an oral side esophagostoma on the left side of the neck, and jejunostomy of the feeding route.

Second-stage surgery is additional cardiovascular surgery if needed. In case 2, the distal anastomotic site of the TAR was considered to be the main infection site associated with the fistula, so TEVAR was performed for reinforcement. In case 4, as in case 1, the main site of infection associated with the fistula was at the junction between two prostheses; in this case, between the proximal end of the DAR (installed 17 months earlier) and the distal end of the TAR (installed 50 days earlier). However, infection also caused a pseudoaneurysm at the distal anastomosis of the DAR, so in stage 2 the DAR was performed again. In case 1 and 3, no additional surgery was done there.

Third-stage surgery is esophageal reconstruction. This was performed in cases 1, 2, and 3, after improvement of general conditions and infection findings. In those 3 cases, a gastric tube was formed using the patient’s stomach to replace the esophagus, in an antethoracic route.

### 2.3 Outcomes after staged surgery

Case 1 survived the third-stage surgery, but suffered hypercalcemia associated with a tumor and renal failure, and died on day 224 of hospitalization. Case 2 recovered and was discharged from our hospital on day 227. In case 3, after esophagus reconstruction, suture failure occurred between the esophagus and the gastric tube, and formed a skin fistula. As of day 216 of hospitalization, she has received conservative treatment for that problem. Case 4 survived after second-stage surgery, but has continued to receive treatment for infection and chronic disseminated intravascular coagulation. As of day 160 of hospitalization, esophageal reconstruction has not been achieved.

## 3 Discussion

All the AEF cases described here occurred after prosthetic aortic replacement for thoracic aortic aneurysm. These fistulae were formed at the contact between the distal anastomotic site of TAR and the middle thoracic esophagus. One possibility is that the esophagus fistula, perhaps caused by ischemic necrosis with compression (that is, with mechanical stimulation or irritation) or operative technique, leaked esophageal contents onto the aortic prosthesis, caused infection there. Alternatively aortic prosthetic infection might have occurred first, perhaps due to caries, hemodialysis, or invasive treatment, and that infection led to the esophageal fistula. Xi et al. suggested that stent graft infection should be considered as the main mechanism of AEF formation [11]. In our 4 cases the progress after first surgery and the elapsed times from surgery to onset varied. Identifying the causes of AEF in any consistent manner is difficult.

Some papers suggested that combined surgery provided better outcomes for some patients of secondary AEF [[7], [8], [9]] (Table 2). What they suggested in common is that the first treatment of secondary AEF is infection control, with esophagectomy being the first stage. The subsequent timing of second- and later-stage surgery must be judged by a comprehensive assessment of the degree of improvement with infection control and nutritional status. There is the question of whether to replace an infected prosthetic graft. There have been reports of an infective thoracic aortic aneurysm and artificial stent graft being removed and the aorta restored in second-stage surgery [8,9]. These cases involved the first thoracotomy, whereas our cases involved the second one. This is a large difference. Infection may be controlled by esophagectomy and antibiotic treatment, so we think that prosthesis replacement is not always necessary. However, experience has shown that where a prosthetic graft meets the native aorta, infection brings a real danger of aneurysm. In case 2, TEVAR was done in the second stage as a precaution, because the native aorta remained at the infected site.

**Table 2:** Summary of strategies for secondary aortoesophageal fistula.

Year	Author [Reference]	Number of cases	Surgery of fistula	Outocomes
2012	A. Saito et al. [4]	Six cases (four after TEVAR and two after open graft replacement)	Five cases received staged surgery and one received simultaneous it.	Two in-hospital deaths (one case of staged surgery and one of simultaneous it)
2012	M. Amano et al. [7]	One case after TAR	Three staged surgery (first: esophagectomy and esophagostomy, second: re-replacement, Third: esophageal reconstruction)	Discharge
2013	H. Munakata et al. [8]	One case after TEVAR	Two staged surgery (First: aortic replacement, esophagectomy and esophagostomy, Second: retrosternal gastric bypass)	Discharge
2019	A. Kamigaichi et al. [9]	One case after TEVAR	Three staged surgery (first: esophagectomy and esophagostomy, second: aortic replacement, Third: esophageal reconstruction)	Discharge

In case 1, 2, and 3, a gastric tube was used to replace the esophagus, and it was implanted in an antethoracic route. One reason for this choice was the fact that anastomotic failure and cutaneous fistula formation occurred in all of 3 cases who underwent esophageal reconstruction. In the cases of AEF after prosthetic replacement, the upper or middle part of the esophagus is damaged, and the esophagostoma to be formed in the first stage is on the clavicle. The third-stage reconstruction has to bridge an unusually great distance, and we suspect that resulting mechanical stress and inadequate blood-flow led to anastomotic failure. Reconstruction methods need to be considered, for example selecting colon reconstruction.

## 4 Conclusion

We reported the clinical features and outcomes of treatment of secondary AEF after prosthetic aortic replacement in 4 cases. Based on our experience, the approach involving early diagnosis, disruption of the infected fistula by esophagectomy, treatment of prosthetic graft infection, prevention of possible pseudoaneurysm formation, and esophageal reconstruction, appears to be a valid therapeutic strategy for secondary AEF.

## Funding

The authors declare there are no funding resources for this paper.

## Ethical approval

Institutional review board approval was not required because all data were collected from clinical records and imaging systems for routine preoperative planning and follow-up.

## Consent

Written informed consent was obtained from all of the patients for publication of this case report and accompanying images. A copy of the written consent is available for review by the Editor-in-Chief of this journal on request.

## Author contribution

ME wrote the manuscript. TK, NT, and TS supervised writing the manuscript. All authors were part of the surgical team that treated these patients. All authors read and approved submission of the final manuscript.

## Registration of research studies

We have registered our research at http://www.researchregistry.com. The unique identifying number of our study is “researchregistry5919”.

## Guarantor

Masahide Enomoto.

Tomoaki Suzuki.

## Provenance and peer review

Not commissioned, externally peer-reviewed.

## CRediT authorship contribution statement

**Masahide Enomoto:** Data curation, Writing - original draft. **Takeshi Kinoshita:** Visualization. **Noriyuki Takashima:** Visualization. **Fumihiro Miyashita:** Data curation. **Tomoaki Suzuki:** Supervision, Project administration, Writing - review & editing.

## Declaration of Competing Interest

The authors report no declarations of interest.
